# Changes in Collagen Structure and Permeability of Rat and Human Sclera After Crosslinking

**DOI:** 10.1167/tvst.9.9.45

**Published:** 2020-08-31

**Authors:** Peng Guo, Yuan Miao, Yang Jing, Sruti Akella, Fang Wang, Choul Yong Park, Cheng Zhang, Roy S. Chuck

**Affiliations:** 1 Department of Anatomy and Structural Biology, Albert Einstein College of Medicine, Bronx, NY, USA; 2 Analytical Imaging Facility, Albert Einstein College of Medicine, Bronx, NY, USA; 3 Gruss-Lipper Biophotonics Center, Albert Einstein College of Medicine, Bronx, NY, USA; 4 Department of Ophthalmology and Visual Sciences, Albert Einstein College of Medicine, Montefiore Medical Center, Bronx, NY, USA; 5 Aier School of Ophthalmology, Central South University, China; 6 Department of Ophthalmology, Dongguk University Ilsan Hospital, Goyang, South Korea

**Keywords:** crosslinking, collagen structure, sclera, second-generation harmonic imaging, two-photon excited fluorescence microscopy, fluorescence recovery after photobleaching

## Abstract

**Purpose:**

To use second harmonic generation imaging and fluorescence recovery after photobleaching to demonstrate alterations in scleral collagen structure and permeability after crosslinking in rat and human eyes.

**Methods:**

Excised rat and human scleras were imaged ex vivo with an inverted two-photon excitation fluorescence microscope before and after photochemical crosslinking using riboflavin and 405-nm laser light. Fluorescence recovery after photobleaching was applied to measure the diffusion of fluorescein isothiocyanate–dextran across the sclera.

**Results:**

Crosslinking caused scleral collagen fibers to become wavier and more densely packed, with surface collagen being more affected than deeper collagen fibers. Crosslinked sclera showed significantly decreased permeability in the irradiation zone and also extended as far as 250 µm outside the irradiation zone.

**Conclusions:**

Photochemical crosslinking induced changes in scleral structure and permeability that extended to tissue even outside the irradiation zone.

**Translational Relevance:**

Ultrastructural changes associated with the emerging clinical technique of photochemical scleral crosslinking have not been well characterized. We demonstrate not only changes in scleral collagen by second harmonic generation imaging but also the associated functional changes in tissue permeability by fluorescence recovery after photobleaching. We report the novel finding of reduced permeability extending well beyond the direct irradiation zone. This has implications for control in the clinical setting.

## Introduction

Collagen and elastin are the two major components of the conjunctiva, Tenon's capsule, and sclera.^1^ Collagen fibers form collagen bundles, which are grouped into layers of collagen lamellae.^2^ Conventional imaging of these tissues has been performed with histologic examination, immunohistochemistry, and electron microscopy.^3^ However, the tissue fixation process and labeling dyes may disrupt the natural architecture of these delicate tissues, limiting our understanding of in vivo structure.

Second harmonic generation (SHG) microscopy is a well-established technique to study collagen morphology. It has several advantages over conventional methods. It allows the examination of collagen fibrils without exogenous dyes, because collagen is an efficient generator of second harmonic radiation signals under two-photon excitation. Therefore, no staining is required to observe the collagen, making it possible to image in vivo with minimal invasiveness. In addition, near-infrared excitation wavelengths used in SHG imaging allow deeper imaging depth, greater than 100 µm because of less scattering.^4^

Recent studies have used SHG to further elucidate corneal collagen structures in normal corneas and in disease states such as keratoconus.^5^^,^^6^ Others have used SHG to examine the effects of photochemical crosslinking (CXL), an indicated treatment for keratoconus and other kerectasias, on collagen structures in cornea.^4^^,^^7^ Our approach, then, offers a quantitative characterization and understanding of sclera diseases.

Fluorescence recovery after photobleaching (FRAP) is a powerful microscopy method that reveals the diffusion dynamics of fluorescently tagged molecules within live tissues. In this context, FRAP can be used to measure scleral permeability. As is well known, a decrease in scleral permeability may be related to an increase in tissue stiffness, leading to greater risk of glaucoma.^17^ Fewer studies have used SHG to examine collagen structure and arrangement in conjunctiva and sclera,^8^^,^^9^ and only one has done so after CXL.^10^ In this study, we used a combination of SHG and FRAP to determine (1) how collagen fibrils in the sclera change after CXL and (2) whether permeability is affected.

## Materials and Methods

This study was approved by the Institutional Review Board and Institutional Animal Care and Use Committee of the Albert Einstein College of Medicine and adhered to the tenets of the Declaration of Helsinki.

### Sample Preparation

Rats were euthanized by overdose with carbon dioxide, and the eyes were harvested for further experiments. Both eyes of each rat were placed in phosphate-buffered saline (PBS) and cleaned of extraocular tissues. The scleral tissue was excised 1.8 mm away from the limbus at the equator (1 mm above and below the equator) between muscle insertions, as tissue closer to the limbus was difficult to flatten after CXL for microscopy. Human eye bank cornea–sclera rim buttons (14 days from death to use; Saving Sight Eye Bank, Kansas City, MO) were stored in Optisol GS (Bausch & Lomb, Rochester, NY) solution until imaged. These buttons were classified as “research only” for various reasons (mild corneal endothelial pleomorphism/polymegathism, diffuse moderate endothelial stress lines, guttata) but were maintained in an optically transparent state. The cornea–sclera buttons were transferred to a Petri dish, and several drops of balanced salt solution (Alcon, Fort Worth, TX) were applied to the samples to prevent desiccation. Then, 4-mm-round punched scleral tissues were excised immediately next to the limbus from the eye bank tissue.

The dissected rat/human scleral tissues were placed in 0.5% riboflavin solution for 30 minutes and washed by PBS twice. They were then placed in fluorescein isothiocyanate (FITC)–dextran solution (FD-40, molecular weight 40 kDa; Sigma-Aldrich, St. Louis, MO), at a concentration of 1 mg/mL in PBS and incubated in the dark for 20 minutes. Samples were mounted on a glass-bottom culture dish with the outer sclera side facing down and cover-slipped with an additional 30 µL of 0.5% riboflavin solution. A smaller area of rat or human sclera, 512 × 512 µm was selected for CXL under the microscope (half of this area was crosslinked with laser, the other half served as non-CXL control).

### Second Harmonic General Imaging Process

Second harmonic generation imaging was performed using an inverted Olympus IX81 two-photon excited fluorescence microscope (FluoView FV1000; Olympus Corp., Central Valley, PA). The imaging process was, briefly, as follows: The cornea–sclera section was placed on a glass-bottom plate (35 mm; Thermo Fisher Scientific, Waltham, MA). A two-photon laser (Mai Tai DeepSee Ti:Sapphire oscillator; Spectra-Physics, Santa Clara, CA) was tuned to 850 nm and directed through a red dichroic mirror (690 nm), and a water immersion objective (25×, 1.05 numerical aperture [NA]) was used to focus the excitation beam and to collect backscatter SHG signals. The signal was collected through a bandpass emission filter (425/30 nm) after reflection by a dichroic mirror (458 nm), and a square image (500 × 500 µm) was acquired with 1024 × 1024 pixels of resolution in approximately 15 seconds; multiple image slices (*z*-stacks) were acquired using the same objective lens. When images were *z*-stacked, samples were scanned in 1-µm steps in the *z*-axis to generate three-dimensional datasets. For reference, the 0-µm position in depth corresponds to the first *z*-position where the SHG signal was detected.

### Ex Vivo Crosslinking

Scleral tissue was crosslinked via standard 405-nm confocal laser scanning on the same inverted Olympus IX81 two-photon excitation microscope. The microscope was equipped with a continuous-wave, 405-nm diode laser (Melles Griot, Rochester, NY). The laser beam was approximately 500 nm in diameter on the sample through a 25×, 1.05-NA water immersion objective. A 512 × 512-µm region of interest (ROI) was scanned via a 1024 × 1024-pixel frame format with a dwell time of 20 µs pixel per image frame. The scanning was performed with 100 iterations. The laser power was controlled by FluoView software. The 405-nm laser power ranged from 0.4 mW to 1.22 mW as measured by a power meter and was used for crosslinking.

### Fluorescence Recovery After Photobleaching

In principle, FRAP is performed by photobleaching fluorescent molecules at a specified location in tissue and monitoring the rate at which the bleached molecules are replaced by unbleached ones.^23^ The rate of recovery reflects the rate of movement of the fluorescently tagged molecules at that location within the tissue. Here, the FRAP technique was used to measure the diffusion of FITC–dextran molecules. Measured diffusion is an indicator of permeability of small molecules in the scleral tissue:

(1) $$I_{norm}\;\left(t\right)=\frac{I\left(t\right)-I_{bkg}\left(t\right)}{I_{prebleach}-I_{bkg}\left(t\right)}\;\times \frac{T_{prebleach}-I_{bkg}\left(t\right)}{T\left(t\right)-I_{bkg}\left(t\right)}$$

where *I* is the intensity of fluorescence signal at time *t*, *I_bkg_* is the background intensity, *I_prebleach_* is the intensity prior to photobleaching, and *T* is the intensity of unbleached region far away used as a reference in the image. The recovery curve is fitted by an exponential function to extract the half time of the recovery curve:

(2) $$I\;\left(t\right)=\left(1-e^{-t/\tau }\right)\;+I_{0}$$

(3) $$t_{1/2}=\;-\frac{\ln0.5}{\tau }$$

FRAP imaging data were collected using the same Olympus IX81 microscope equipped with a 500- to 530-nm bandpass filter and a XLPlan N, 25×/1.05 WMP water objective lens; the argon 488-nm laser was set at 5% power. The imaging window was 512 × 512 µm, and the bleach zone was a circular disk 15 µm in diameter. Data collection consisted of three pre-scans excited at 5% 488-nm laser power, followed by 100 iterations of 100% 405-nm laser power directed at the bleach zone and subsequently by 78 more full scans taken at 5% 488-nm laser power. Images were centered at the midpoint between the inner and outer sclera. In each region, six bleaching zones were created and results recorded. The intensities of the bleached regions of interest in the images were measured by the ImageJ (National Institutes of Health, Bethesda, MD) plugin FRAP Profiler.

### Statistical Analysis

Paired *t*-tests were performed to compare regions of interest, and *P* values (two-tailed) less than 0.05 were considered significant. Statistical analyses were performed using SPSS Statistics 20.0 (IBM, Armonk, NY).

## Results

### Collagen Structure and Effects of Crosslinking on Rat Sclera

#### Rat Sclera Was Imaged at Different Depths Away from Surface of the Tissue Before Crosslinking

**Figure 1.**
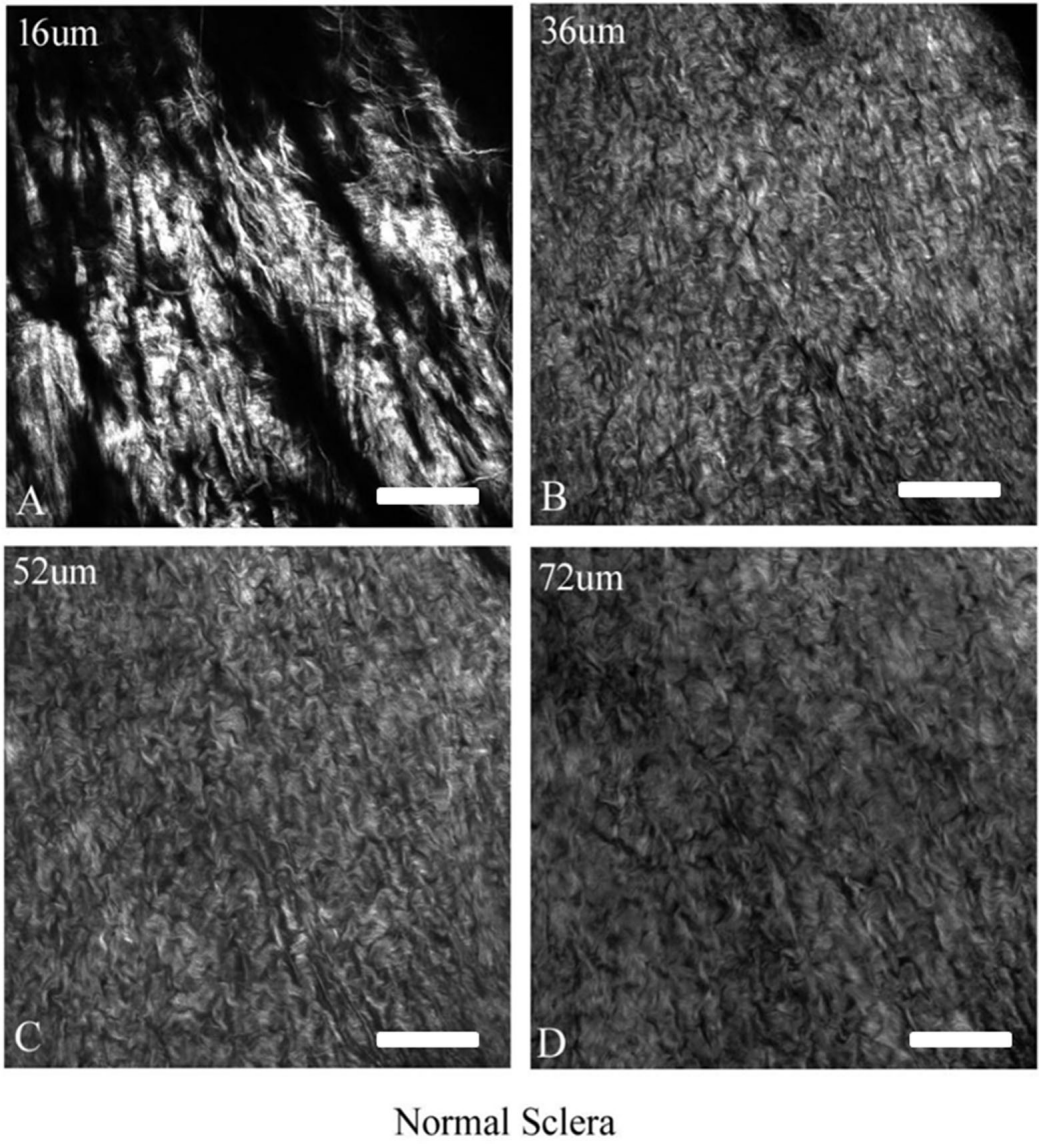
SHG microscopy of rat scleral tissue at different depths. A depth of 0 µm indicates the surface of the scleral tissue where the SHG signal was detected. The thickness of rat sclera is reported to be ∼104 µm.^24^ *Scale bar*: 100 µm.

In Figure 1, before crosslinking, scleral tissue (depth at 16 µm) showed scattered, irregularly arranged collagen bundles and fibrils (Fig. 1A). In deeper sclera (36 and 52 µm deep), collagen appeared to be more densely packed with small, interweaving curled and wavy collagen bundles (Figs. 1B, 1C). In even deeper sclera (72 µm), the wavy collagen bundles were larger and less densely packed (Fig. 1D). Considering the different morphology of collagen at different depths, we were careful to always image at the same 50-µm depth in the following FRAP permeability studies.

#### After Collagen Structure of Sclera Was Imaged, Crosslinking Was Induced by 405-nm Laser Excitation

**Figure 2.**
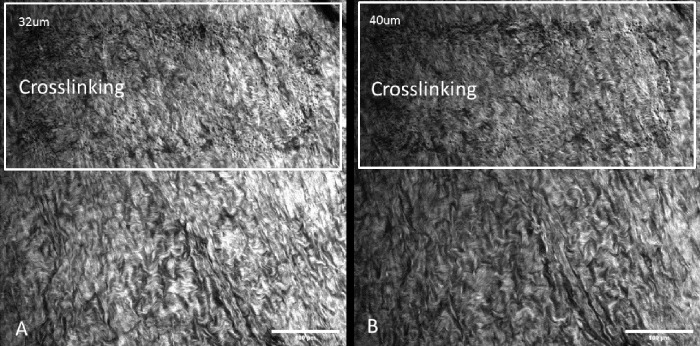
SHG images of rat scleral tissue after crosslinking irradiation at different depths: (**A**) 32 µm and (**B**) 40 µm. In both panels, the top half had undergone crosslinking irradiation. The bottom half shows non-irradiated neighboring tissue for comparison.

In the riboflavin-soaked tissue after crosslinking irradiation, scleral collagen bundles became more disorganized but also more closely packed (Fig. 2). The bundles showed increased homogeneity resulting in a more regular signal pattern than in the untreated zone. This finding of more densely packed fibrils is consistent with the hypothesis that crosslinking decreases the permeability of tissue.

### Permeability After Crosslinking in Rat Sclera

#### Permeability of Sclera Tissue Was Quantitatively Studied by FRAP Before and After Crosslinking Treatment

**Figure 3.**
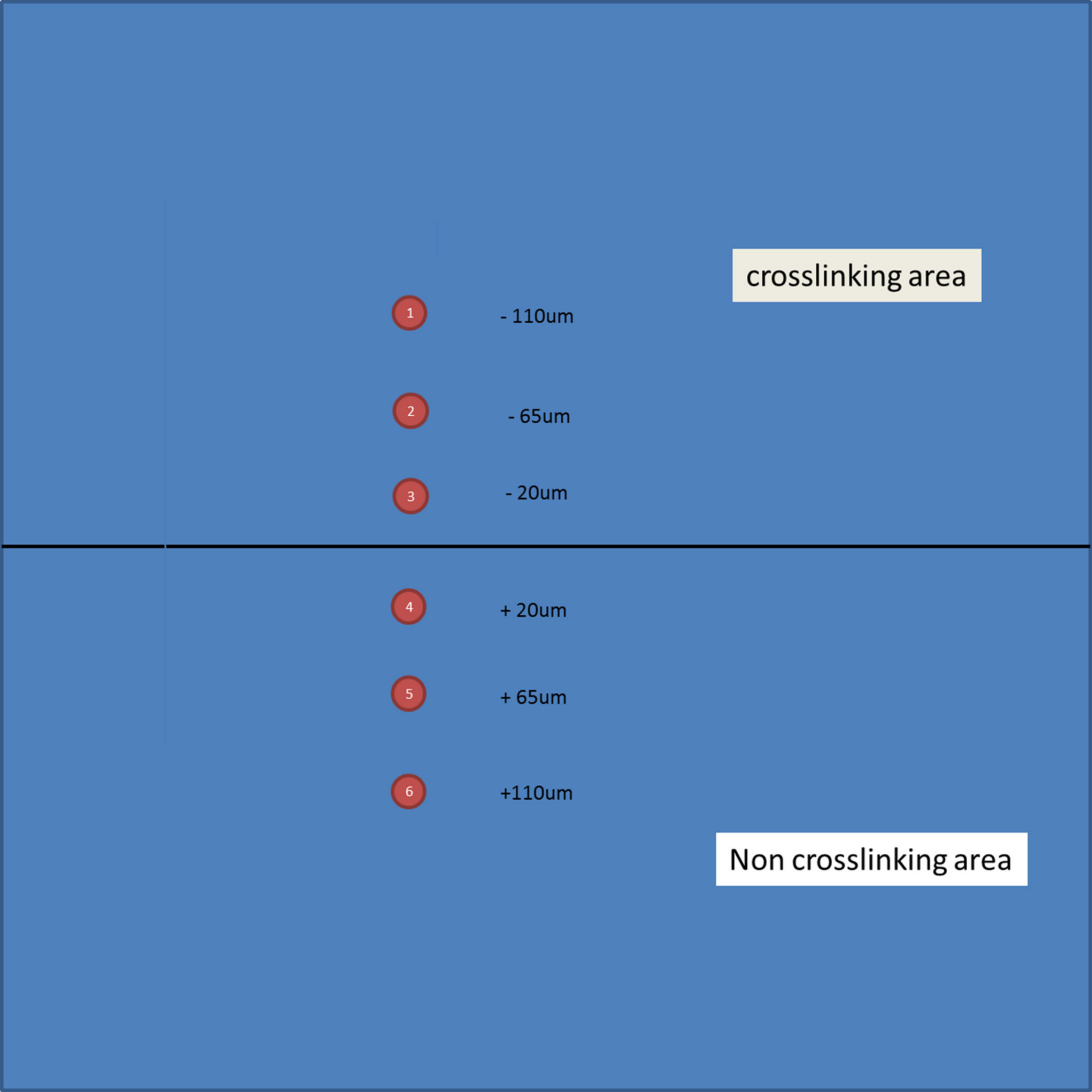
A schematic map showing the locations of six ROIs in which diffusion times were measured. Each ROI is 15 µm in diameter. The photochemical crosslinking area is the top half of the region. The entire image frame is 512 × 512 µm.

**Figure 4.**
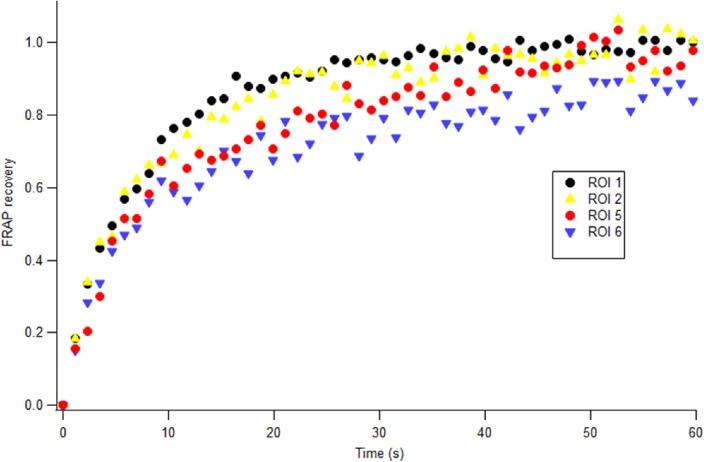
Fluorescence intensity recovery curves in various ROIs in the rat scleral tissue from Figure 3. Only four representative curves are shown for clarity. Variability in the shape and slopes was clearly observed in the recovery curves, demonstrating differences in small particle diffusion in crosslinked versus non-crosslinked tissue.

**Figure 5.**
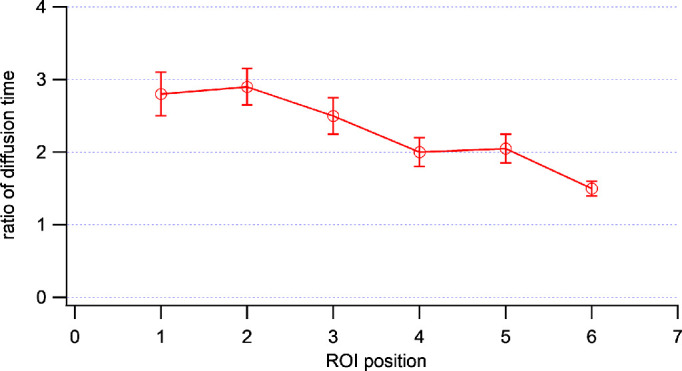
Changes in diffusion times after crosslinking at each ROI shown in Figure 3, normalized by the control samples’ diffusion times before crosslinking in rat sclera. Four rat tissue samples from four rats were used in the study.

The recovery curves show qualitatively the change in the speed of diffusion of small molecules such as fluorescein inside sclera. To further understand the effect of crosslinking, we performed curve fitting to the curves to quantitatively determine the change in diffusion time across crosslinked and non-crosslinked zones in sclera tissue. We defined a parameter referred to as the ratio of diffusion time, which is the value of the diffusion time at each location in the tissue in Figure 3 divided by that of non-treated, non-crosslinked tissue. This value is a quantitative measure of how much the permeability property of the tissue is altered from crosslinking. As shown in Figures 4 and 5, the rat sclera tissue displayed significantly increased diffusion time inside the irradiation area (ROIs 1 to 3), as the diffusion time was over twice that in non-irradiated area. However, this statistically significant decline pattern also persisted farther away from the localized area of irradiation (Figs. 3, 4). Even outside the direct irradiation area, the diffusion time still displayed a ratio greater than 1 at ROIs 4 to 6 compared to non-treated tissue.

### Crosslinking Laser Energy Titration

**Figure 6.**
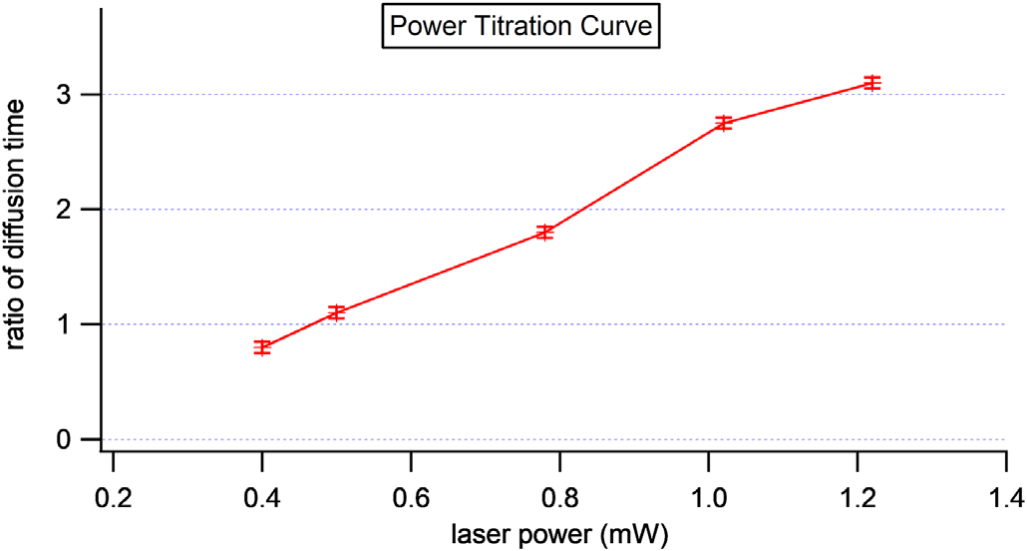
Changes in diffusion time due to varying the stimulus of a 405-nm laser.

We also studied the power response of a 405-nm stimulus laser for crosslinking. After gradually increasing the input power from 0.4 mW, we found that the ratio of diffusion time was significantly increased above 1 when the energy input level was above 0.5 mW (second data point in Fig. 6) and scan time was 20 µs/pixel, iteration 100. This finding established the proper laser dosage for inducing permeability change in sclera. It also provides a reference for inducing crosslinking.

### Permeability after Crosslinking in Human Sclera

**Figure 7.**
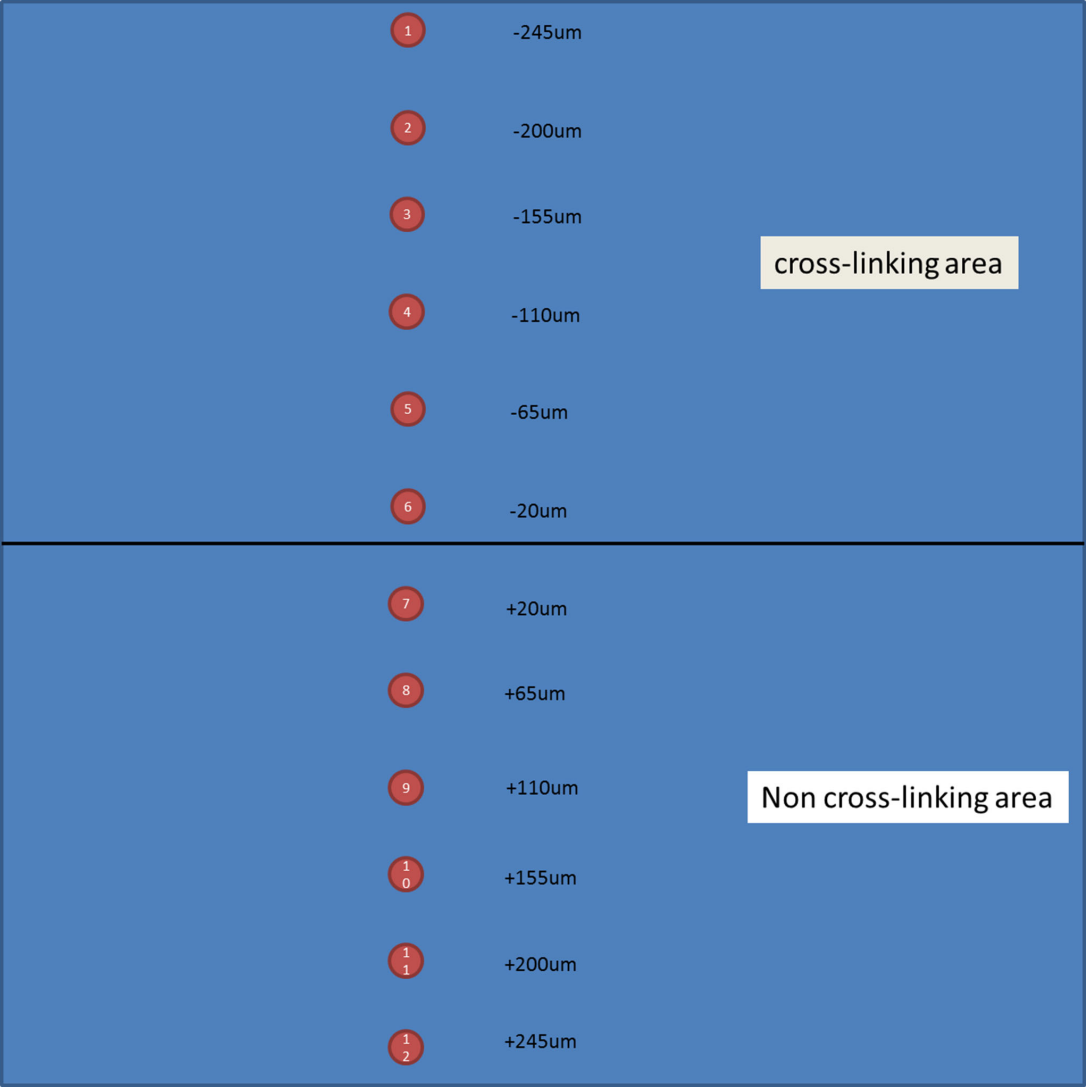
A schematic map showing the locations of 12 ROIs in human sclera at which diffusion times were measured. The entire image frame is 512 × 512 µm.

**Figure 8.**
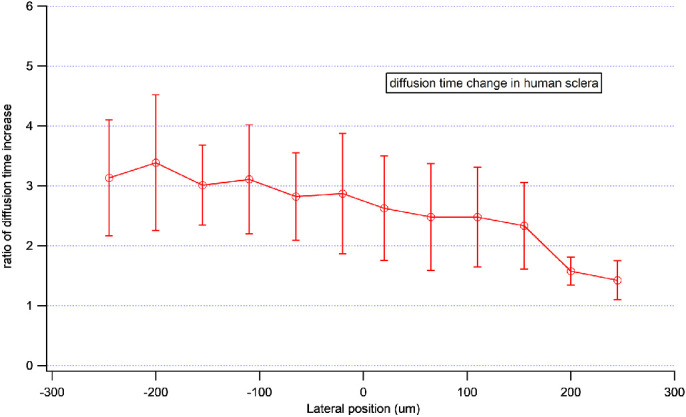
Normalized diffusion time changes in the irradiation area and adjacent tissue as shown in Figure 7. This experiment was repeated three times.

Crosslinking was also performed in human eye bank sclera. FRAP was used to measure the diffusion of FITC–dextran in ROIs at varying locations inside and outside of the irradiation area (Fig. 7). Each ROI represents a circular area 15 µm in diameter and 45 µm from the previous and subsequent ROIs. As shown in Figures 7 and 8, diffusion time was predictably high in irradiated crosslinked sclera, indicating a decrease in permeability. More striking is the observation that the diffusion time decreased at distances farther from the treatment area, and overall neighboring tissue was more permeable than untreated sclera; that is, the transition zone within the sclera was not sharply demarcated at the sharp irradiation beam boundary. The curve shows a gradual decline of diffusion/permeability at ROIs further away from the irradiation area. The decline only reached a ratio close to 1 at ROI 12, which was 25 µm away from the irradiation boundary. We tested for changes in preservation medium (Optisol) for 1 day versus 1 week and found no significant changes in either the SHG images or FRAP recovery measurements. Additionally, preservation medium was washed out before microscopy. We performed CXL and SHG imaging immediately after mounting tissue samples in their dishes. The entire process consistently took less than half an hour in all cases. Because additional incubation time with riboflavin might cause additional CXL effects, we took great effort to make the incubation period consistent in each experiment.

## Discussion

Photochemical crosslinking was approved by the Food and Drug Administration in 2016 for the treatment of keratoconus and post-refractive surgery corneal ectasia. There has been growing interest in clarifying the molecular structural and biomechanical changes that occur after treatment, and such investigations have conventionally been done using light and electron microscopy. Xia et al.^11^ used polarized light microscopy and observed a microscopically detectable corneal haze with a significant increase in type III collagen fibrils between 30 and 90 days after crosslinking therapy with riboflavin and ultraviolet light. Karl et al.^22^ used electron microscopy to examine how the fibrils change after crosslinking and found similar changes in structure with blue light treatment.

As second harmonic generation imaging has become more widespread, a few studies have used it to examine the microscopic effects of crosslinking on corneal collagen structure.^12^^–^^14^ Steven et al. found homogeneous patterns in cornea after chemical crosslinking. It was also reported by the Vukelic group^21^ that crosslinking locally using a femtosecond laser increases the density of collagen structures in the anterior layers of the stroma, resulting in a homogeneous signal pattern more regular than that of the untreated cornea, similar to our findings in rat sclera. Another group also reported an increase in the stiffness of rabbit corneas after nonlinear laser pulse illumination.^27^ We found that crosslinking introduced a more densely packed structure of collagen in rat and human sclera tissue, which was visually distinctive compared to untreated areas.

We further quantitatively examined the permeability of tissue after crosslinking using FRAP, a technique in which the diffusion dynamics of fluorescently tagged molecules are tracked over time. Three previous studies have applied this technique to scleral tissue.^15^^–^^17^ When Kimball et al.^17^ used glyceraldehyde to crosslink the sclera in an attempt to build a mouse model of glaucoma, they found that after glyceraldehyde treatment FRAP time was increased and permeability decreased. This is consistent with our studies in rat and human sclera, although previous studies did not quantify the effects outside of the treatment zones. Additionally, the sclera also became stiffer as the FRAP time increased, with significant retinal ganglion cell loss in their proposed glaucoma model. Scleral permeability of large molecules is also important to the transscleral delivery of therapeutic molecules for the treatment of retinal diseases.^25^^,^^26^

In addition to its quantitative investigation of diffusion properties, our study (performed for the first time in human tissue, to the best of our knowledge) did not identify an abrupt transition zone, despite the use of a single, 500-nm diameter laser beam with high accuracy to irradiate a sharply demarcated area. Rather, the permeability appeared to change gradually over 250 µm (Fig. 8). Such a transition zone is consistent with the work of the Scarcelli group^20^ on porcine cornea; they found a 610-µm transition zone between fully photochemically crosslinked and non-crosslinked sections. The Vukelic group^21^ also found a transition zone in pig cornea after femtosecond laser crosslinking in the range of hundreds of micrometers. The likely mechanism is light scattering that occurs during crosslinking irradiation. There may also exist a flow of free radicals that causes crosslinking outside the treatment area. This finding is relevant clinically; for example, relatively sharp transitions in the cornea may cause more visual aberrations, and gradual transitions, such as we observed, may cause fewer aberrations.^19^

More recently, crosslinking of scleral tissue has been proposed as a mechanism to treat myopia, although the data are limited.^10^^,^^18^ It is important to understand how the sclera reacts to crosslinking. Here, we observed that the effects of crosslinking on permeability persist at a significant distance away from the treatment area. To the best of our knowledge, no previous study has performed an ex vivo examination of the spatial distribution of permeability of human or rat sclera after riboflavin-induced photochemical crosslinking of the sclera. Our FRAP method offers a tool to study this spatial distribution.
